# Complete Genome Sequence of a New Zealand Mycobacterium tuberculosis Strain Responsible for Ongoing Transmission over the Past 30 Years

**DOI:** 10.1128/mra.00781-22

**Published:** 2022-10-13

**Authors:** Claire V. Mulholland, Gregory Gimenez, Adele Williamson, Mackenzie Steele, Duncan Thorpe, Noel Karalus, Ray T. Cursons, Veronica M. Playle, Sally A. Roberts, Gregory M. Cook, Vickery L. Arcus, Htin Lin Aung

**Affiliations:** a School of Science, University of Waikato, Hamilton, New Zealand; b Department of Pathology, Dunedin School of Medicine, Dunedin, New Zealand; c Department of Microbiology and Immunology, School of Biomedical Sciences, University of Otago, Dunedin, New Zealand; d Department of Pathology, Waikato Hospital, Waikato District Health Board, Hamilton, New Zealand; e Department of Molecular Medicine and Pathology, University of Auckland, Auckland, New Zealand; f LabPlus, Auckland City Hospital, Auckland District Health Board, Auckland, New Zealand; g Maurice Wilkins Centre for Molecular Biodiscovery, University of Auckland, Auckland, New Zealand; Wellesley College

## Abstract

We report here the complete genome sequence of Mycobacterium tuberculosis strain Colonial S-type 1 (CS1), which has been responsible for ongoing outbreaks of tuberculosis in New Zealand over the past 30 years. CS1 appears to be highly transmissible, with greater rates of progression to active disease, compared to other circulating M. tuberculosis strains; therefore, comparison of its genomic content is of interest.

## ANNOUNCEMENT

Tuberculosis (TB) is a treatable disease, but it still claims over 1.5 million lives annually (1). Molecular typing of Mycobacterium tuberculosis isolates circulating in New Zealand shows that the Colonial S-type 1 (CS1) strain, formerly known as the Rangipo strain, represents the largest cluster of isolates (2, 3). Phylogenetically, CS1 belongs to sublineage L4.4.1.1 of the Euro-American lineage of M. tuberculosis, and it has been the cause of ongoing outbreaks in the New Zealand population for at least the past 30 years (4, 5). This warrants further studies to characterize its genetics in order to understand its spread and virulence. Here, we report the complete genome sequence of the New Zealand strain CS1, which was isolated in 1996 from a cerebrospinal fluid sample as part of routine clinical diagnosis and strain typing. Ethical approval was not required for this purpose or sequencing of New Zealand clinical M. tuberculosis isolates.

This isolate was recovered from a frozen culture stock and cultured in modified 7H9 medium with Middlebrook oleic acid-albumin-dextrose-catalase (OADC) growth supplement (10%) using the BACTEC mycobacterial growth indicator tube (MGIT) system. DNA was extracted using a cetyltrimethylammonium bromide (CTAB) extraction protocol adapted from reference 6. Briefly, the culture was heat inactivated in Tris-EDTA (TE) buffer, and the pellet was then treated for 2 h at 37°C with lysozyme and RNase A, followed by overnight digestion with proteinase K at 56°C. DNA was then extracted by phenol-chloroform extraction, followed by CTAB-chloroform extraction and isopropanol precipitation (6).

The full-length CS1 genome was sequenced with an RS II (P6-C4 chemistry) instrument (Pacific Biosciences [PacBio], Menlo Park, CA, USA) using SMRTbell libraries with an additional size selection step with the 20-kb-template BluePippin size selection system (Sage Science, Beverly, MA, USA). Paired-end 250-bp short-read sequencing was performed on a MiSeq platform (Illumina, Inc., San Diego, CA, USA) using the Nextera XT DNA library preparation kit (Illumina).

We obtained 157,670 long reads and 1,280,644 paired-end short reads with the PacBio and Illumina systems, respectively. Long-read lengths ranged from 35 to 51,700 bp, with an *N*_50_ value of 7.852 kb. Read quality control was performed using FastQC. Long reads were first trimmed and error corrected (17,152 reads, yielding 153,575,068 cleaned bases) and then assembled using Canu (version 1.8) (7), which resulted in the assembly of a single large circular contig. This contig was polished and error corrected with Pilon (version 1.23) (8) using Illumina short reads, with a mean coverage of >50×. Canu was run with the following options: genomeSize=4.4M, minReadLength=500, and minOverlapLength=200; Pilon was run with default parameters.

The resulting circular genome has a length of 4,416,671 nucleotides, with a GC content of 65.6%, and was annotated using Prokka (version 1.14) (9) with H37Rv as a reference genome(GenBank accession number NC_000962.3). A total of 4,135 coding sequences were found, together with 56 tRNAs and 3 rRNAs. The CS1 strain and H37Rv proteomes share 2,918 proteins with strong homology (>80%). As a reference genome, the CS1 sequence reported here will be helpful in further studies to elucidate the genetic bases for the transmission of this New Zealand strain and its virulence characteristics and host specificity.

### Data availability.

The whole-genome sequence of New Zealand M. tuberculosis strain CS1 has been deposited in DDBJ/ENA/GenBank under the accession number CP044345. The raw reads were deposited in the NCBI Sequence Read Archive (SRA) under the BioProject accession number PRJNA573545 and the SRA accession numbers SRR20995191, SRR20998128, SRR20998236, and SRR20998249.
